# Postauricular injection in the treatment of all-frequency and high frequency descending sudden hearing loss: A protocol for systematic review and meta-analysis

**DOI:** 10.1097/MD.0000000000023847

**Published:** 2021-01-22

**Authors:** Ying Li, Jiao Liang, Han-Jen Chiang, Yang Liu

**Affiliations:** a Department of Rehabilitation Sciences, Faculty of Education, East China Normal University, Shanghai; b Hospital of Chengdu University of Traditional Chinese Medicine, Chengdu, Sichuan Province; c College of Traditional Chinese Medicine, Three Gorges University & Yichang Hospital of Traditional Chinese Medicine, Yichang, Hubei Province, P.R. China.

**Keywords:** all-frequency descending sudden hearing loss, high frequency descending sudden hearing loss, postauricular injection

## Abstract

**Background:**

Sudden hearing loss (SHL) is a disease, at the same time a symptom, which needs to be treated in a timely manner and counts as an emergency health problem in the Department of Otolaryngology. There are many types of sudden hearing loss and among them, the occurrence of all-frequency descending sudden hearing loss and high frequency sudden hearing loss are high. The conventional treatment for these 2 types of sudden hearing loss sometimes is not as effective as expected. Postauricular injection of glucocorticoids could be the most effective treatment method. However, the effectiveness and safety of postauricular injection of glucocorticoid needs to be assessed systematically.

**Methods:**

The protocol for the meta-analysis was conducted under the guidance of Preferred Reporting Items for Systematic Review and Meta-Analysis Protocols (PRISMA-P). The aim is to undertake a systematic review and meta-analysis on the effectiveness and safety of postauricular injection of glucocorticoid to treat patient diagnosed with all-frequency and high frequency descending sudden hearing loss. We searched through the following databases: English databases (PubMed, EMBASE, Web of Science) and Chinese databases (CNKI, Wanfang databases, CBM, VIP). The final selected articles will be evaluated using Cochrane RCT evaluation criteria. Revman 5.0 will be used for data analysis. Subgroup analysis, sensitivity analysis, and meta regression will detect sources of heterogeneity. Ethics approval was not required for this protocol. The findings will be disseminated through journal articles or conference presentations.

**OSF registration number:**

DOI 10.17605/OSF.IO/5Q9NA

**Results:**

Objectively, evaluate the efficacy and safety of postauricular injection of glucocorticoid in treating all-frequency descending sudden hearing loss and high frequency sudden hearing loss.

**Conclusion:**

To provide evidence-based medicine for glucocorticoid treatment methods in patients with all-frequency descending sudden hearing loss and high frequency descending hearing loss.

## 1 Introduction

Sudden hearing loss, also known as sudden deafness, refers to a sensorineural hearing loss that occurs suddenly in one or both ears within 72 hours; presented ≥30 dB at 3 consecutive frequencies, and the cause is unknown.^[1]^ The incidence of sudden hearing loss has increased every year and was thought to be a disease that could self-heal and no special treatment was required.^[2]^ Sudden hearing loss at the range of 250 to 500 Hz is diagnosed as low-frequency sudden hearing loss, around 1000 Hz is diagnosed as middle frequency sudden hearing loss, above 4000 Hz refers to high frequency sudden hearing loss, hearing loss at all frequencies refers to flat-type sudden hearing loss, and extreme hearing loss at every frequency refers to complete/profound deafness^[3]^ The type of sudden hearing loss focused in this paper is all-frequency descending sudden hearing loss and high-frequency sudden hearing loss.

Many clinicians mentioned that postauricular glucocorticoids injection may be one of the most effective treatment method for sudden hearing loss.^[1,4,5]^ Glucocorticoids could suppress the immune response of the patient, adjust the inner ear microcirculation of the patient, and then produce the corresponding therapeutic effect, and reduce the situation of membranous labyrinthus hydrops.^[6]^ Moreover, there are many ways glucocorticoids could be administered: intratympanic injection, sustained release of glucocorticoids into the round window membrane, and postauricular injection.^[7]^ According to many clinicians, administer glucocorticoids through postauricular injection could avoid side effects from the whole body administration and avoid blood-labyrinth barrier blocking glucocorticoids from reaching the ear.^[6,7]^ The purpose of this study is to answer the question whether postauricular injection could effectively treat all-frequency descending sudden hearing loss and high frequency sudden hearing loss. Therefore, a meta-analysis of the effectiveness of glucocorticoid injection behind the ear is necessary.

## 2 Methods

The meta-analysis protocol was conducted under the guidance of PRISMA-P and registered on the OSF platform. The registration number is DOI 10.17605/OSF.IO/5Q9NA.

### 2.1 Search strategy

Two authors will search English database and Chinese database, independently. The databases PubMed, EMBASE, and Web of Science will be searched with English. The databases included CNKI, Wanfang database, CBM and VIP will be retrieved with Chinese. Subject words and free words will be searched with “and,” “or” and “not.” We will search these databases from the time of establishment of the database to November 15, 2020. The search formula is follows: (“Injection” OR “Injectables” OR “Injectable” OR “Zhu She”) AND (“Sudden Deafness” OR “Hearing Loss, Sudden” OR “Sudden Hearing Loss” OR “Tu Fa Xing Er Long” OR “Tu Long” OR “Er Long” OR “Long”) AND (“trial” OR “clinical trial”).

#### 2.1.1 Inclusion criteria

(1) The included population is patients with all-frequency or high frequency sudden hearing loss; regardless of the course of the disease, sex, age, and race.
(2) The intervention methods are postauricular injection of glucocorticoid and conventional treatment.
(3) The treatment of the control group would be conventional treatment.
(4) Observation indicators include effective rate, comparison of all-frequency or high-frequency hearing threshold, and incidence of adverse reactions.
(5) The study of research is randomized-controlled trial (RCT). If the number of RCT research articles is <7, non-RCT researches can also be included.

#### 2.1.2 Exclusion criteria

(1) If the patients have any of the following diseases, they would be excluded from the research: acoustic neuroma, inner ear malformation, middle ear disease, hereditary deafness, mental disorder, severe diabetes, and hypertension.
(2) Those who are contraindicated to glucocorticoid use will be excluded.
(3) Patients during pregnancy and lactation will be excluded.
(4) Abstracts, letters, case reports, reviews or nonclinical studies will be excluded.
(5) Besides conventional treatment, the experimental group and the control group cannot have other intervention methods, otherwise the study will be excluded.
(6) Research on data reuse will be excluded.

### 2.2 Article retrieval process

**Figure 1:**
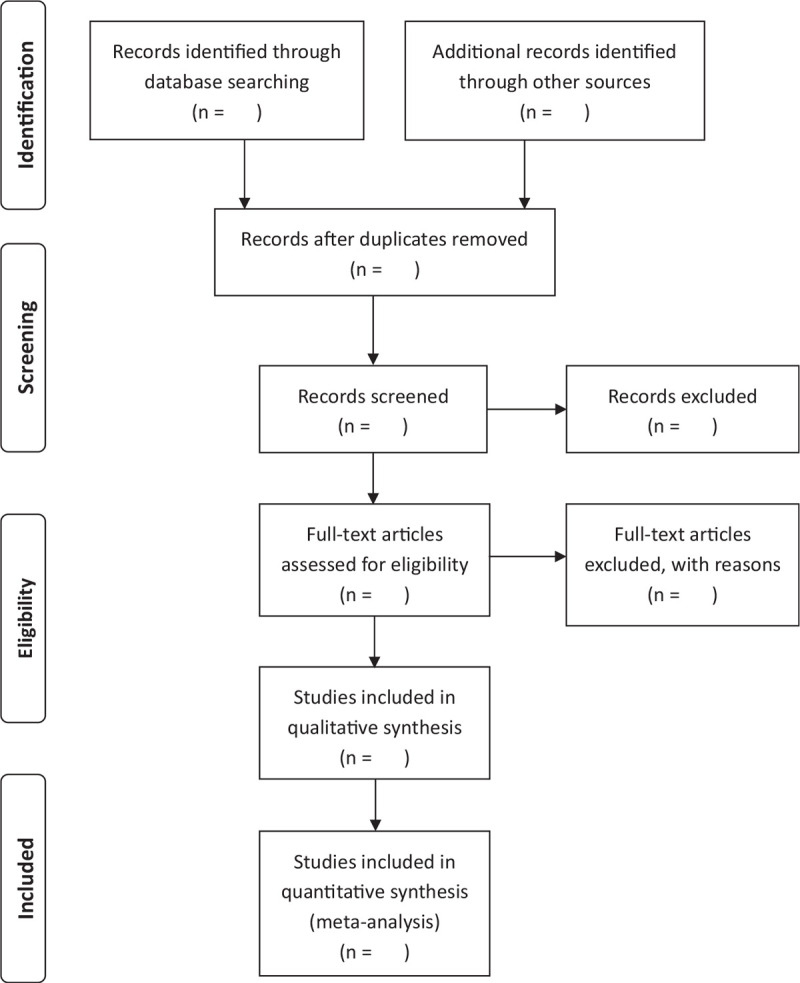
Flow diagram of the identification and selection studies.

Two authors will independently select articles according to the flowchart shown below (Fig. 1). If there is a dispute during the screening process, an additional author will be added to discuss the final included article.

### 2.3 Article quality evaluation

Different research types require different evaluation methods. The Cochrane Risk Bias Assessment Tool is used to evaluate the quality of research of RCT. Two authors will use Revman 5.0 software (Nordic Cochran Centre, Copenhagen, Denmark) to evaluate the quality of the article, independently. If there is a disagreement, a third author will be added to discuss together and finally determine the quality of the article. The final results of the article quality evaluation are presented in the form of graphs.

### 2.4 Data extraction

Two authors extracted the data independently. If there is a disagreement, the third author will join and discuss the finalization of the extracted content. The information extracted from all selected articles will be stored in an Excel table and appear in the article in the form of a table (Table 1). The storage information is as follows: first author, publication year, study type, patient age, sex, intervention measures, control measures, effective rate, full frequency or high frequency hearing threshold changes, incidence of adverse reactions, etc.

**Table 1:** Main characteristics of the studies included in the meta-analysis.

Basic information of articles	Authors, publication year
Country of publication	Country
Design	Research type (RCT or non-RCT)
Gender	Male/Female (Number in each group)
Age	Age in each group
Interventions	Treatment for each group
Effective Rate	Effective rate in each group
Audiogram	Full frequency or high frequency hearing threshold changes in each group
Incidence of adverse reactions	Adverse reaction rate in each group

#### 2.4.1 Data analysis

The data analysis will be based on the number of articles included. If the number of included articles is ≥7, we will conduct a meta-analysis. If the number of included articles is <7, we will only make a descriptive systematic review. We will use Revman5.0 software for meta-analysis. The analysis includes: combined effect size, heterogeneity test, publication bias, etc. Among them, subgroup analysis, sensitivity analysis, and meta regression will be used to detect the source of heterogeneity. *Q* test and *I*^2^ test are used to detect the size and degree of heterogeneity. When *P* < .10 or *I*^2^ ≥ 50%, it indicates that there is significant heterogeneity. Relative risk is used as the effect scale of effective rate and the incidence of adverse reactions. Weighted mean difference/standard mean difference is used as the effect scale of full-frequency or high-frequency hearing threshold changes. When combining effect size, if *P* < .05, the combination is effective.

#### 2.4.2 Subgroup analysis

Subgroup analysis is used to explore the source of heterogeneity. Criteria for subgroup classification can be based on clinical characteristics, such as article quality, audiogram characteristics of sudden deafness (all-frequency drop or high frequency drop), length of illness, and conventional treatment plan. In subgroup analysis, the groups are homogeneous and merged into heterogeneity. This factor is the source of heterogeneity.

#### 2.4.3 Sensitivity analysis

Changing the analysis model and excluding articles one by one is the content of sensitivity analysis. When *I*^2^ > 50%, the random effects model is selected. If *I*^2^ < 50%, the fixed effects model is selected. When excluding the articles one by one, it is found that the heterogeneity has changed significantly; the excluded article is the source of the heterogeneity.

#### 2.4.4 Meta-regression

In Meta-regression analysis, the dose of the drug injected behind the ear, the patient's age, the patient's sex, and the course of disease can be used as covariates to find the source of heterogeneity. When *P* < .05, it indicates that this factor is the source of heterogeneity.

#### 2.4.5 Publication bias

For publication bias, we will use a funnel chart to identify it. When there is publication bias, the funnel chart is skewed, asymmetry appears.

## 3 Discussion

According to the frequency and degree of hearing loss, it is usually divided into high frequency descending type, low frequency descending type, all-frequency descending type, and total deafness type.^[8,9]^ Among them, the high frequency descending type refers to the frequency above 2000 Hz (inclusive) hearing loss ≥20 dB, the all-frequency descending type hearing loss refers to the 250 to 8000 Hz frequency range average hearing threshold ≤80 dB, and the total deafness type refers to the 250 to 8000 Hz frequency range average hearing threshold ≥81 dB.^[10]^ Mainstream medicine believes that different types of hearing curves suggest different pathogenesis. Among them, the all-frequency descending type is mostly striated vascular disease or inner ear vasospasm, the high frequency descending type is mostly hair cell damage, and the total deafness is inner ear vascular embolism or thrombosis formed.^[11]^ It is also advocated that the medications for different hearing curve types should be different. Medications for the high frequency hearing loss type and the all-frequency descending type (including the total deaf type) hearing loss include hormone medication combined with microcirculation improvement drugs and neurotrophic drugs.^[18]^ So, these 2 types of hearing loss are discussed together.

Glucocorticoids are the preferred treatment for sudden hearing loss, also known as sudden deafness, and the medication methods mainly include systemic medication and topical medication.^[12–14]^ According to relevant research results, injections behind the ears can effectively avoid secondary effects caused by systemic medication; symptoms such as insomnia, persistent hiccups etc. Therefore, glucocorticoids injected behind ears are applicable to a larger area than systemic medication. For example, it can be applied to patients with diabetes, gastric ulcers, etc.^[15–17]^ Behind the ear injections can effectively avoid side effect caused by intratympanic injections like tympanic membrane perforation, otitis media, balance sensory disturbances, etc. Owing to the related reasons, some scholars think inject glucocorticoids behind ear is safer than tympanic injection.^[1]^ Some scholars believe that behind the ears injection may have its own unique drug onset mode, not only it can avoid the blood-labyrinth barrier, but also allow the drug concentrated at the cochlear. Compared with other methods, it is higher and more stable, and it's time to reach the cochlea is shorter, and its curative effect may be better.^[18,19]^ In clinical practice, more and more doctors use hormone injection behind the ear as an effective treatment for sudden deafness.^[18,20]^ Especially for patients with sudden deafness who didn’t have a positive response to conventional treatments, it can be used as a salvage treatment. Based on many clinicians’ observation, it is indeed effective. The “Healing and Treatment Guidelines” in the “Diagnosis of Sudden Deafness” published by Chinese experts in 2015 recommended that glucocorticoid injection behind the affected ear as a remedial treatment to treat sudden hearing loss.^[20]^

There may be 4 mechanisms for the postauricular injection of glucocorticoids.^[21]^ The first is through the endolymphatic sac; it is found that there is a close connection between the sigmoid sinus and the endolymphatic sac, and the blood supply system of the endolymphatic sac is dual blood supply.^[22]^ Drugs injected behind the ear can enter the endolymphatic sac through the sigmoid sinus and the occipital artery to take effect.^[22]^ The second path is through anatomical fissures; bone fissures and sieve area are examples of the natural fissures. It is possible for drugs to enter the middle ear through the fissures, which can then diffuse to the inner ear to play its role.^[22]^ The third is through systemic circulation route; the drug injected behind the ear is absorbed into the blood, enters the internal jugular vein and the subclavian vein, and then enters the brain from the systemic circulation system to nourish the inner ear and exert its effect.^[22]^ The last method is through the stylomastoid artery; which provides blood supply to the inner ear, and the injection of drugs behind the ear can exert its effect through it.

According to relevant literatures, it is believed that the treatment effect of all-frequency descending hearing loss and high-frequency descending hearing loss is not good enough. Injecting glucocorticoid behind the affected ear(s) seems to bring hope for these 2 types of sudden deafness patients, but it has not been obtained evidence-based support yet, so it is necessary to conduct a meta-analysis and systematic analysis of the efficacy of glucocorticoid injection behind the ear for these two types of sudden hearing loss. We will try our best to collect all data and conduct an objective evaluation of this.

## Author contributions

**Conceptualization:** Ying Li, Yang Liu.

**Data analysis:** Ying Li, Jiao Liang.

**Data curation:** Jiao Liang, Han-Jen Chiang.

**Formal analysis:** Jiao Liang, Han-Jen Chiang.

**Funding acquisition:** Yang Liu.

**Methodology:** Jiao Liang, Han-Jen Chiang.

**Software:** Jiao Liang.

**Study design:** Yang Liu, Ying Li.

**Supervision:** Yang Liu.

**Writing – original draft:** Ying Li.

**Writing – review & editing:** Ying Li, Yang Liu.
